# Clinical Relevance and Psychometric Properties of the Swedish Version of the Cultural Competence Assessment Instrument

**DOI:** 10.1155/2020/2453239

**Published:** 2020-04-04

**Authors:** Jane Holstein, Gunilla M. Liedberg, Yolanda Suarez-Balcazar, Anette Kjellberg

**Affiliations:** ^1^Department of Health, Medicine and Caring Sciences, Faculty of Medicine and Health Sciences, Linköping University, Linköping, Sweden; ^2^Department of Occupational Therapy, University of Illinois at Chicago, Chicago, USA

## Abstract

Based on the increasing diversity of Swedish society, health professionals, like occupational therapists, find it challenging to provide culturally competent services to international clients. Consequently, cultural competence among professionals needs to be measured and improved using psychometrically tested instruments. This study examines the clinical relevance, construct validity, and reliability of the Swedish version of the Cultural Competence Assessment Instrument among Swedish occupational therapists. *Material and Methods*. A randomised sample of 312 Swedish occupational therapists answered a survey based on the Swedish version of the Cultural Competence Assessment Instrument with supplementary questions on the clinical relevance of the instrument. Descriptive statistics were used to examine the clinical relevance of the Swedish version of the Cultural Competence Assessment Instrument. Factor analyses, both exploratory and confirmatory, were run to examine the factor structure. Cronbach's alpha was performed to assess the internal consistency of the instrument. *Results*. The participants reported that the 24 items had high clinical relevance. The validation yielded a three-factor model: openness and awareness, workplace support, and interaction skills. All three of these factors showed high loadings. *Conclusions*. The study results indicated positive clinical relevance and psychometric properties for the Swedish version of the Cultural Competence Assessment Instrument and strong support to be utilised in Sweden. The implications of this study are important given the rapid growth in migration over the last few decades. A self-rating instrument measuring cultural competence could support occupational therapists' professional knowledge and development when they interact with international clients. As the tool was originally developed in English in the United States, the feedback from the Swedish version could potentially be useful for the instrument in modified form and for use by occupational therapists in English-speaking countries.

## 1. Introduction

Many immigrants face challenging health issues, which puts pressure on the healthcare system to provide culturally relevant interventions [1]. The Swedish Migration Agency [2] has reported that more than one million people fled to the European countries in 2015 to seek protection from persecution and war. Never before have so many people seeking protection in Sweden as in 2015. Immigrants originate from many different locations around the globe, and they respond to trauma, migration stressors, and resettlement difficulties in a variety of ways, based on cultural backgrounds, personal characteristics, and healthcare experiences [3]. Occupational therapists and other health professionals need to adapt and meet the changing demands and demographic characteristics of the general population [4]. These health professionals, including occupational therapists, must therefore have the cultural competence to meet the needs of a more diverse group of clients than they are accustomed to dealing with [1]. Given the challenges that practitioners may face in addressing the needs of diverse populations, practitioners may find it useful to have access to tools that enable them to examine their perceived level of cultural competence [5].

Cultural competence can be defined as the professionals' understanding of how culture affects their views and activities as well as the interventions that they apply [6]. Cultural competence can also be viewed as a contextual and dynamic process where health professionals have the ability to meet the needs of the clients with understanding and efficient communication, regardless of their client's background, ethnicity, and/or cultural nuances [7, 8]. Cultural competence also implies demonstrating cultural humility, where health professionals are willing to learn and are open to new ways of working. Other similar terms have been used in the literature to refer to cultural competence, such as culturally responsive care, cultural awareness, and cultural sensitiveness [9]. For consistency, we will use the term “cultural competence.”

## 2. Cultural Competence Assessment Instrument

The Cultural Competence Assessment Instrument (CCAI) is developed in United States [7] and has been culturally adapted and translated to Swedish. It is referred to as The Cultural Competence Assessment Instrument-Swedish version (CCAI-S). CCAI-S is a self-reported instrument that is completed by the therapist for measuring his/her own cultural competence. The content validity and utility of CCAI-S have been evaluated in a qualitative study [10]. The results indicated positive content validity for the CCAI-S and its potential to be utilised in the Swedish context. The results revealed that all 24 items in the CCAI-S were considered valid, although six items needed clarifications. The results also specified important aspects regarding utility of the instrument. CCAI-S had potential for use in diverse workplaces and in teams with diverse professions. It showed the importance of organisational support in the improvement of communication and cultural competence. The CCAI-S could also be utilised individually to raise awareness about cultural topics in everyday practice [10].

The theoretical basis for the instrument is the conceptual model of cultural competence for occupational therapists and other health professionals and was developed by Balcazar et al. [7] and Suarez et al. [11]. The model starts with an elementary assumption that health professionals have the desire to serve, meet the needs, and learn about individuals from diverse populations. The model comprises three domains. The cognitive domain mirrors the professional's own critical awareness and comprehension and involves a multifaceted progression of self-reflection. This awareness begins with the readiness to question one's own views and then adjustment to strategies from their understandings of the client's culture. The behavioural domain comprises evolving skills such as verbal and nonverbal communication. This domain involves the professionals' efforts to effectively and empathetically communicate with the client, as well as efforts to integrate the opinions, values, experiences, and determinations of the person during the therapeutic process. The contextual domain emphasises support performing multicultural practices from the organisation or work setting. Organisational support (support from the work setting) is vital for practitioners to engage in cultural competent practice.

The construct validity of the original version of the CCAI is based on factor analysis in a random sample of 477 occupational therapists. It showed strong psychometric support for the 24 items and the three factors [11]. One study used the CCAI to examine perceived cultural competence in a sample of 477 American occupational therapists [8]. It showed that prior training, both formal and informal, was positively correlated with higher levels of cultural competence.

Cultural competence among occupational therapists needs to be measured and improved, by using psychometrically tested instruments. Therefore, it is crucial to develop reliable, valid methods for assessing cultural competence. The initial evaluation of the validity and utility of the CCAI-S was a first step in developing the instrument [10]. To further strengthen the validity and utility of the CCAI-S, it is necessary to investigate in a larger randomised sample of occupational therapists. Hence, the purpose of this study was to examine clinical relevance, construct validity, and reliability of the CCAI-S among Swedish occupational therapists.

## 3. Methods

### 3.1. Sample Selection and Procedure

From a database of 6174 registered occupational therapists available from the Swedish Occupational Therapy Association, a stratified random sampling generated 1116 participants for this study. Two inclusion criteria were used. The first was occupational therapists that worked in the working areas that included ≥1% of the registered occupational therapists in the database. The second was occupational therapists who in their clinical practice had met international clients during the last year. A web-based survey link was sent via email to 1116 occupational therapists followed by three reminders. In addition, a fourth reminder was sent out as a postal survey to respondents' home addresses and included a cover letter, the survey, and a stamped return envelope. Altogether, 427 respondents answered, giving a total response rate of 38%. Of these, 312 respondents completed the survey, i.e., they had met international clients in the last 12 months. Furthermore, 115 respondents answered seven questions in section one regarding demographics and in addition one question on clinical relevance: “Would you consider a self-assessment instrument that measures cultural competence among occupational therapists useful?” These 115 respondents had not met any international clients the last year. The data collection took place between April 2017 and August 2017.

### 3.2. Ethical Consideration

The study involved no psychological or physical risk to participants, and no data regarding the participants' private conditions was collected; thus, formal ethical approval was not required [12]. The declaration of the Helsinki protocol [13] was governing confidently, and informed consent was followed. All participants were provided with written information about confidentiality of collected data, and that raw data would be kept in a secured locked drawer. An opportunity to ask questions of the researchers was provided.

### 3.3. Data Collection

The CCAI-S and an information letter were distributed to the participants. The first section of the CCAI-S focuses on seven demographic questions regarding gender, education, current work setting and occupation, years of practice, and the professional's continent/country of birth. The next section involves nine questions on experiences, the most common languages spoken by the professional's clients, continents of origin of the clients, previous training in cultural competence, and perceived level of cultural competence in relation to clients from different continents. The last section comprises 24 self-rating questions designed to measure three factors each containing eight items: (1) cultural awareness and knowledge; (2) cultural skills; and (3) organisational support for multicultural practice. A six-point Likert-style scale was used, where six is “strongly agree” and one is “strongly disagree.”

The present study also assessed utility, i.e., the application of the assessment tool in everyday practice. One aspect of utility is clinical relevance, which is measured in this study [14, 15]. Each of the 24 items had a supplement utility question: “How relevant is the statement for assessing cultural competence?” In addition, three utility questions were formulated. The first question—“After completing the assessment, how can the occupational therapist utilise the instrument in their encounters with international clients?” This question was divided into four subquestions according to the occupational therapy process. The second question—“After completing the assessment, how can the teams/workgroups/colleagues utilise the instrument in their encounters with international clients?”—gave the respondents the opportunity to choose multiple options such as to identify educational needs in the group. The last question asked respondents to rate the following question—“After completing the assessment, what utility may the organisation have when staff have utilised the instrument in their encounters with international clients?” The participants could choose among multiple options, such as to provide support for educational efforts and skills development. The three supplemental utility questions were answered on a four-point Likert type scale where 1 was equivalent to the lower attribution (i.e., not relevant, not useful) and 4 was equivalent to the highest positive attribution (i.e., very relevant, very useful).

### 3.4. Statistical Data Analysis

Statistical analyses were performed with the statistical package IBM SPSS Statistics and AMOS (version 24.0; IBM Inc., New York, USA). Descriptive statistics were used to analyse demographic data and the utility questions. Chi-squared tests were used to analyse the responses to the question on the clinical relevance of the assessment tool during the preceding year. The level of significance was set at *p* < 0.05 using Fisher's exact test [16] and where the cell size was below 5. In the study, the utilised CCAI-S had to be >50% to be considered positive. To determine the construct validity of the instrument, exploratory factor analysis (EFA) was used identifying the need for a minimum sample size of 72 [17]. A total of 312 respondents completed the survey, but 24 surveys were eliminated due to missing responses, yielding a total of 288. To test sample adequacy, the Kaiser-Meyer-Olkin (KMO) test was applied; a KMO above 0.60 is an acceptable value [16]. The sample (*n* = 288) was randomly divided into a subsample (*n* = 144) as described by Bollen [18] and Dragioti et al. [19]. EFA using the principal component extraction method with varimax rotation was applied to the subsample [16, 20, 21]. In addition, Bartlett's test of sphericity was applied to investigate whether the correlations were equal across the samples. If the test displayed a significant level of 0.05 or lower, the items are equally correlated and will ensure the possibility to perform a factor analysis (FA) [16].

The use of the EFA confirmed the reduction of data categories down to a three-factor model containing 12 items. The factors were given labels based on the weighted combinations of the items in each factor. Through the use of confirmatory factor analysis (CFA) with a *p* < 0.05, the inconsistency reliability coefficients were calculated [16].

## 4. Result

### 4.1. Demographic Data of the Participants

Table 1 presents the characteristics of the participants in the two groups: those who in the previous year had met international clients (*n* = 312) and those who had not met international clients (115) in the previous year but still had responded on the question of usefulness of a self-assessment instrument regarding cultural competence.

### 4.2. Clinical Relevance

The participants confirmed the 24 items were relevant to their understanding of cultural competence.  Table 2 presents the clinical relevance and psychometric statistics that identified 90–96% of the participants. Five of the eight items in “cultural awareness” were found to be very relevant/relevant. Two items were rated at 85–89% as very relevant or relevant, and the remaining one was rated as very relevant or relevant by 77% of the participants. Of the respondents who had served international clients the last year, 79% reported that an instrument that measured cultural competence would be useful. Of the respondents who had not met international clients the last year, 80% reported that an instrument that measured cultural competence would be useful.

In the factor organisational support for multicultural practice, there was a varied distribution between the eight items regarding the clinical relevance. One item was considered very relevant/relevant by 91% of the participants, four items were rated very relevant/relevant by 80-89% of the participants, and three items were rated very relevant/relevant by 71-77% of the participants. One item—“My workplace does not support my participation in my clients' cultural celebrations”—was rated very relevant/relevant by 53% of the participants. Three of the eight items in the factor cultural skills were rated as very relevant/relevant by 90-95% of the participants. The remaining five items were rated as very relevant/relevant by 81-87% of the participants.

Participants were asked to rate the usefulness and importance of several items in three utility questions (Table 3). The first one concerned the clinical relevance of the instrument for occupational therapy practitioners when assessing and formulating goals, implementing interventions, and engaging in an evaluation. Between 72 and 78% of all participants considered CCAI-S very useful or of great use in relation to implementing an intervention. The second one concerned the utility of the CCAI-S for teams and colleagues when interacting with foreign-born persons in clinical practice and was rated as clinically relevant by 78-87% of the participants. The final question concerned the utility for the organisation and 85% of the participants viewed CCAI-S as contributing to more effective meetings and practices in relation to the patients/clients. A lower degree of clinical relevance (64%) was reported for the use of the CCAI-S as contributing to a more effective use of resources such as using interpreters.

### 4.3. Construct Validity

#### 4.3.1. Exploratory Factor Analysis

This test demonstrated construct validity of CCAI-S (*χ*^2^ = 470, 66: DF = 66; *p* = 000). Factor analysis provided a logical explanation of 57% of the variance of the 12 items within three factors. More factors did not increase the explained variance to a considered extent. The three factors in this study were interpreted as follows: factor I was labelled “Openness and awareness,” factor II “Workplace support,” and factor III “Interaction skills.”

*Factor I*. Five items emerged as strongly related and all items had high loadings (0.748, 0.737, 0.696, 0.683, and 0.672). Three items concerned openness such as learning from clients, willingness to discuss with other practitioners, and learning through educational methods and experience. Two items concerned awareness such as examining own biases that influence behaviour and actively strive for self-exploration.

*Factor II*. This factor was labelled Workplace support and four items had high loadings (0.755, 0. 713, 0.554, and 0.513). Two items focused on the support from colleagues in the workplace regarding feedback on practice skills and opportunities to learn cultural competence. Two items reflected how materials, pictures, and goals in the workplace could support cultural competence. Cross-loading was found for one item: “I have opportunities to learn culturally responsive behaviours from peers.” This item also had a factor loading above 0.4 in “Openness and awareness,” but this item was strongly related to the workplace environment and opportunities to learn cultural competence in terms of the conceptual fit.

*Factor III*. This factor was labelled “Interaction skills.” Three items were jointly loaded in this factor with the highest loadings on all items in a factor (0.774, 0.772, and 0.701) compared to the items in factors I and II. Two items in the factor focused on verbal and nonverbal communicative skills, and one item focused on how easy it was to work competently with clients.

#### 4.3.2. Confirmatory Factor Analysis

The three-factor model proposed in the EFA was confirmed by the CFA showing absolute fit (*χ*^2^ and degrees of freedom = 1.646). The goodness of fit index threshold was set at 0.95, which was again confirmed (0.953), as were the standardized root mean square residual (0.05) and root mean square error of approximation (0.47) [18, 22]. The comparative fit index (0.95) was acceptable, as was the adjusted goodness of fit (0.95) [18, 22]. The three-factor model had a good *p* of close fit (0.57) [18, 22] (see Table 4).

In addition, the three-factor model demonstrated associations among the three latent variables. Between the factors “Openness and awareness” and “Workplace support,” the association was 0.62, and correlation concerning “Openness and awareness” and “Interaction skills” was 0.60. The correlation between “Workplace support” and “Interaction skills” was 0.51. These correlations were in line with the simple item correlations.

### 4.4. Reliability

The internal consistency of the instrument as a whole was supported by Cronbach's alpha, 0.81, while the internal consistency of each of the three factors was demonstrated by Cronbach's alpha coefficients as follows: factor I “Openness and awareness,” 0.79; factor II “Workplace support,” 0.64; and factor III “Interaction skills,” 0.69. These levels were acceptable [16].

## 5. Discussion

The emphasis of the current study was on instrument development in terms of examining the clinical relevance [14, 15], construct validity, and reliability [21] of the CCAI-S, among a randomised sample of Swedish occupational therapists. In a previous study of the CCAI, the construct validity of the original CCAI was tested through FA in a random sample of 477 occupational therapy practitioners in the United States, and it showed strong psychometric support for all three factors and 24 items [11]. The factor analysis in this study showed three factors: “Openness and awareness,” “Workplace support,” and “Interaction skills.” These factors were confirmed by previously performed factor analysis, although labelled somewhat differently compared to the American version of CCAI: “Cultural awareness and knowledge,” “Organisational support for multicultural practice,” and “Cultural skills” and with less items in the factors.

Based on the psychometric misfit, the present study revealed that 12 of the 24 items should be removed in the CCAI-S. In the factor “Openness and awareness,” the majority of the items were considered clinically very relevant or relevant by most of the participants. Based on the factor loadings lower than 0.4, three of these items should be removed. Two of the items were emphasised as difficult to understand in the qualitative study [10] examining validity and utility and the findings from the two studies supported the removal of the two items. The item “I am sensitive to valuing and respecting differences between my cultural background and my clients' cultural heritage” could be removed based on the factor loadings. However, this item was rated almost maximum clinically relevant by the participants and can be regarded as the most important first step for cultural awareness. Based on this reasoning concerning conceptual fit [23], the item will remain in the upcoming published version.

As described by Darawsheh et al., being culturally aware and prepared is accompanied by an open attitude and a respect for cultural differences and is essential if cultural competency is to be achieved and actualised in practice [24]. Furthermore, two items in factor openness and awareness imply discussions with others and examine own biases. This is in accordance with what Beagan [25] describes as critical reflexivity on disparities and inequalities. Reflecting, being critical and discussing with colleagues may contribute to cultural competence for the occupational therapist.

In the factor “Workplace support,” the eight items related to the clinical relevance varied in distribution. Based on the FA, four of the eight items should be removed. In the previous qualitative study by Holstein et al. [10], the participants requested that two of the items have examples and be clarified. These two items, supported by the results of the qualitative study [10] and this study, will be excluded in the upcoming published version. The factor “Organisational support” had previously not been included in a validated cultural competence instrument before CCAI [11]. Organisational support seems to be a vital reason for defining the capacity of the healthcare practitioner to deliver culturally relevant services, since practitioners do not function in a vacuum [7]. In relation to one item, mission statements in the workplace are relevant in this context. If diversity and inclusion efforts are not communicated as a central part of the organisation's mission statement and integrated into day-to-day actions, the mission statement will be ineffective [26]. Another included item focuses on equipment in the workplace, such as pictures and printed materials. In a study by Kjellberg et al. [27], it was found that organisational and financial problems were barriers to being client-centred in occupational therapy practice. Limited financial resources in a workplace can make it more difficult to equip the rooms/spaces with culturally relevant objects and thereby restrict the therapist from performing effective multicultural occupational therapy.

In the factor “Interaction skills,” four items were rated as very relevant/relevant and four items as very relevant/relevant. However, based on the FA, five of these items could be removed. Furthermore, one item overlaps (cross-loading) with another item in the factor “Openness and awareness” and will therefore be excluded here based on the conceptual fit of the item. The other four items are negatively formulated such as “I do not feel” and “it is difficult” and were reported to be difficult to comprehend based on the former study on CCAI-S [10]. This difficulty together with the psychometric misfit means five items will be deleted in the upcoming published version of the CCAI-S. Two of the remaining items focus on communicative skills, which can be considered as important when interacting with international clients. Taylor [28] described that when establishing a good relationship between the occupational therapist and the client, the communication will be successful if it is straightforward, honest, and comfortable. The third item that will remain focused on the interaction between the therapist and the client. In addition to being important for effective communications, cultural skills involve integrating the beliefs, experiences, and values of the individual [11]. Bonder et al. [29] describe the importance for the therapist of establishing a relationship and trust with clients, which in turn will improve the efficiency with which interventions are conducted. Two of the items for interaction skills focus on effective nonverbal and verbal communication with clients from cultures different from that of the occupational therapist.

The three domains (i.e., factors) of “Openness and awareness,” “Workplace support,” and “Interaction skills” constitute both a personal and environmental perspective on cultural competence. “Openness and awareness” and “Interaction skills” are connected to the individual's way of handling/acting when providing service to a multicultural group of clients. This involves the health professionals' own perceptions/preparedness/skills on how to interact with clients from a culture other than their own. The third domain, “Workplace support,” represents the environmental circumstances present in the workplace. This includes organisational support for multicultural practices, such as policies, resources, work environment, and team members/peers. The personal and environmental perspectives interact dynamically, thus influencing each other and contributing to providing culturally relevant services. This type of interaction parallels what is described in two occupational therapy models [30, 31].

The instrument was rated as having high clinical relevance in relation to occupational therapy practitioners, teams, and the organisation. The majority rated the CCAI-S as useful for making meetings more effective in discussing ways to address the needs of international clients. However, 64% of all participants rated the CCAI-S as low clinical relevance with respect to this question: “Make a more efficient use of existing resources, such as interpreters.” Even though the CCAI-S captures the need for resources such as interpreters, this rating shows that the CCAI-S is less able to influence the more efficient use of resources. It seems that this question on interpreters is connected to a wider issue within healthcare, since the provision of relevant interpreters must be supported by the organisations [7] and must be appropriate in terms of minority languages and gender [32]. Language barriers are obstacles to the provision of therapy and can affect compliance with treatment [33]. Yet, the result indicates that the CCAI-S can be considered as an instrument that may be utilised by health professionals other than occupational therapists. Nevertheless, further studies are needed that include different types of practitioners for establishing the utilisation of the CCAI-S for other healthcare and social services practitioners.

The results identified that some of the questions were not relevant based on the psychometric analysis; nevertheless, they were considered by the respondents as being clinically relevant. This could be explained by the fact that conceptually, all 24 items relate to one another and some items in fact may be similar in concept. The psychometric analysis enabled the researchers to clean the instrument and reduce redundancy yielding a more clear and unique tool. However, one item that had psychometric misfit based on the FA will be kept in the CCAI-S because the participants noted that it was the most clinically relevant item of all the items.

### 5.1. Methodological Considerations

The major strength of this study is the combination of examining clinical relevance and using psychometric analysis when culturally adapting and translating the American version of CCAI into a Swedish version. This has given a reliable and solid ground for further development of the CCAI-S.

The stratified random sampling ensured that representation from the stratified working areas was proportional to the working areas of the whole population in the database of registered occupational therapists in Sweden. Nevertheless, certain limitations must be considered when interpreting the result. The response rate was not as large as the study sample of the American version of the instrument. One reason may be that cultural competence is a new area of research in Sweden. Occupational therapists in Sweden are also not familiar with instruments in the area of cultural competence. Another potential explanation may be that occupational therapists in Sweden have not yet provided service to international clients to the same extent as occupational therapists in United States. The area in question could also be regarded as sensitive since cultural competence can be considered as a critical issue to reflect upon for health professionals. In a Hadziabdic et al. study testing validity and reliability in the Swedish version of measuring cultural awareness in nursing students, the researchers emphasise that it is challenging to acquire consistent items in an instrument when it deals with sensitive and multidimensional issues [34].

The current survey was based on the CCAI-S with supplementary questions on clinical relevance. Although the questionnaire is extensive, it was estimated to take 20-30 minutes to complete, a reasonable amount of time for questionnaires. The advantage of using questionnaires is the complete anonymity for the respondents and the absence of interviewer bias [21]. This psychometric study yielded a validated and translated tool that has strong validity and reliability for use in the Swedish context. Given that CCAI-S was only validated for occupational therapy practitioners, other health professionals should use it with caution. Future studies should include a random sample of health professionals from diverse disciplines, such as nurses, physicians, physiotherapists, and social workers, and further analysis of its application. In addition, future studies should examine CCAI-S's utility in different practice settings, such as in-patient and out-patient rehabilitation and home care, and across the lifespan, i.e., children, youth, young adults, and older adults. Given the global phenomena of migration and refugees searching for a home country free of war, this study of cultural competence has important implications internationally for occupational therapists and other health professionals. The present study can represent a model for international professionals interested in validating and adapting the CCAI to their country and culture.

## 6. Conclusion

The examination of the CCAI-S demonstrates high clinical relevance. The factor analysis yielded a three-factor model similar to the American version concerning the number of factors but with fewer items in each factor. The CCAI-S showed high homogeneity, i.e., Cronbach′s alpha = 0.81. These results indicate a close relationship among the items, so the items fit the concept of cultural competence. Based on a thorough analysis of the results from the clinical relevance questions together with the psychometric fit, 13 items will be included in the upcoming CCAI-S, the first version to be published and used in Sweden. The CCAI-S will give occupational therapists a tool that facilitates an improved approach in cultural competence in meeting a growing diverse Swedish population.

## Figures and Tables

**Table 1:** Demographic characteristics of the participants who responded to the question of usefulness of a self-assessment instrument regarding cultural competence, showing both the group that had met international clients (*n* = 312) and the group that had not met them in the previous year (*n* = 115).

	Met international clients previous year, *n* = 312, *n* (%)	Did not meet international clients previous year, *n* = 115, *n* (%)	*p* value
Gender			1.000
Women	302 (97)	111 (97)	
Men	10 (3)	4 (3)	
Age^1^			0.017
Mean/±SD/range	47.9/11.2/22-73	50.7/9.9/31-66	
Highest education			≤0.001
Research level	7 (2)	18 (16)	
Master level	31 (10)	16 (14)	
Bachelor level	274 (88)	81 (70)	
Working years			0.543
1-10	75 (24)	20 (17)	
11-20	91 (29)	36 (31)	
21-30	93 (31)	38 (33)	
31-	53 (17)	21 (18)	
Working areas^2^			
Geriatric care	66 (21)	25 (22)	
Primary care	47 (15)	3 (3)	
Other^3^	50 (16)	31 (28)	
Regional hospital care	30 (10)	3 (3)	
Vocational rehabilitation	28 (9)	4 (3)	
Paediatric rehabilitation	27 (9)	4 (3)	
Municipal services for people with disabilities	19 (6)	12 (10)	
Psychiatric in patient and out-patient care	24 (8)	3 (3)	
Somatic hospital care	21 (7)	5 (4)	
Education/pedagogical work	8 (3)	17 (15)	
Assistive technological centres	14 (5)	7 (6)	
Social psychiatry	11 (4)	6 (5)	
Adult rehabilitation	11 (4)	5 (4)	
Low vision clinic	9 (3)	2 (2)	

^1^Missing data regarding age (*n* = 1). ^2^The participants had the possibility to fill in more than one alternative. ^3^Other could, for example, consist of partial workplaces and management positions.

**Table 2:** Clinical relevance and psychometric misfit for the 24 items.

Items	Clinical relevance of item Very relevant/relevant (%)
Factor: cultural awareness	
I actively strive for an atmosphere that promotes risk-taking and self-exploration. (Item 4) *n* = 305	93
I examine my own biases related to ethnicity and culture that may influence my behaviour as a service provider. (Item 3) *n* = 305	91
I openly discuss with others issues I have in developing multicultural awareness. (Item 1) *n* = 307	90
I learn about different ethnic cultures through educational methods and/or life experiences. (Item 2) *n* = 307	91
I feel that I can learn from my ethnic-minority clients. (Item 6) *n* = 310	85
Items with psychometric misfit based on the factor loadings below 0.4	
I am sensitive to valuing and respecting differences between my cultural background and my clients' cultural heritage. (Item 5) *n* = 310	96
It is difficult for me to accept that religious beliefs may influence how ethnic minorities respond to illness and disability. (Item 7) *n* = 306	89
I do not consider the cultural backgrounds of my clients where food is concerned. (Item 8) *n* = 304	77
Factor: organisational support for multicultural practice	
Cultural competence is included in my workplace's mission statement, policies, and procedures. (Item 9) *n* = 310	91
I have opportunities to learn culturally responsive behaviours from peers. (Item 16) *n* = 306	80
I receive feedback from supervisors on how to improve my practice skills with clients from different ethnic-minority backgrounds. (Item 14) *n* = 310	77
At work, pictures, posters, printed materials, and toys reflect the culture and ethnic backgrounds of ethnic-minority clients. (Item 13) *n* = 310	74
Items with psychometric misfit based on the factor loadings below 0.4	
My organisation does not provide ongoing training on cultural competence. (Item 11) *n* = 310	89
My workplace does not support using resources to promote cultural competence. (Item 10) *n* = 307	84
The way services are structured in my setting makes it difficult to identify the cultural values of my clients. (Item 15) *n* = 302	71
My workplace does not support my participation in my clients' cultural celebrations. (Item 12) *n* = 300	53
Factor: cultural skills	
I would find it easy to work competently with ethnic-minority clients. (Item 19) *n* = 306	92
I am effective in my nonverbal communication with clients whose culture is different from mine. (Item 18) *n* = 307	90
I am effective in my verbal communication with clients whose culture is different from mine. (Item 17) *n* = 308	85
Items with psychometric misfit based on the factor loadings below 0.4	
I feel confident that I can learn about my clients' cultural backgrounds. (Item 22) *n* = 306	95
I feel that I have limited experience working with ethnic-minority clients. (Item 20) *n* = 305	90
It is difficult to practice skills related to cultural competence. (Item 21) *n* = 299	83
I do not feel that I have the skills to provide services to ethnic-minority clients. (Item 24) *n* = 307	87
It is hard adjusting my therapeutic strategies to ethnic-minority clients. (Item 23) *n* = 308	81

**Table 3:** Clinical relevance questions and answers.

Questions	Answers			
1. After completing the assessment, how can the occupational therapist utilise the instrument in their encounters with international clients? (The respondents could choose one answer.)	Very useful (%)	Great use (%)	Little use (%)	No use (%)
(a) When assessing	12.7 *n* = 300	60.3 *n* = 300	24.4 *n* = 299	2.3 *n* = 300
(b) When formulating goals	15.6 *n* = 301	57.1 *n* = 301	24.6 *n* = 301	2.0 *n* = 301
(c) When planning and implement interventions	18.0 *n* = 300	59.3 *n* = 300	20.3 *n* = 300	1.7 *n* = 300
(d) When evaluating	11.6 *n* = 301	60.1 *n* = 301	25.9 *n* = 301	2.0 *n* = 301
2. After completing the assessment, how can the teams/workgroups/colleagues utilise the instrument in their encounters with international clients? (The respondents could choose multiple answers.)	To identify educational needs in the group (%)	To get an overview of the group's awareness and knowledge about cultural skills (%)	To get a basis for discussion in the group regarding cultural competence and ethnicity in relation to patients/clients (%)	To make the group aware of attitudes and prejudices (%)
	78.0 *n* = 296	87.0 *n* = 297	86.5 *n* = 297	84.5 *n* = 297
3. After completing the assessment, what utility may the organisation have when staff have utilised the instrument in their encounters with international clients? (The respondents could choose multiple answers.)	Provide support for educational efforts and skills development. (%)	Make a more efficient use of existing resources, such as interpreters. (%)	Provide more effective meetings and practices in the practice of staff with patients/clients who can influence assessment, interventions, and evaluations performed in a more relevant cultural patient/client-centred manner. (%)	
	86.2 *n* = 297	64.0 *n* = 297	84.5 *n* = 297	

**Table 4:** Confirmatory factor analysis with absolute and relative fit using three-factor model (12 items) of CCAI-S (*n* = 288).

*C*_MIN_/DF^1^	CFI^2^	GFI^3^	AGFI^4^	SRMR^5^	RMSEA^6^	*P*_CLOSE_^7^
1.646	0.948	0.953	0.948	0.05 (criterion < 0.09)	0.047	0.571

^1^Chi-square (*χ*^2^) and degrees of freedom. ^2^Comparative fit index. ^3^Goodness of fit index. ^4^Adjusted goodness of fit. ^5^Standardized root mean square residual. ^6^Root mean square error of approximation. ^7^*p* of close fit.

## Data Availability

The data used to support the results of this study are available from the corresponding author upon a reasonable request.

## References

[1] Rechel B., Mladovsky P., Ingleby D., Mackenbach J. P., McKee M. (2013). Migration and health in an increasingly diverse Europe. *The Lancet*.

[2] The Swedish Migration Agency (2017). The changing influx of asylum seekers in 2014-2016: Member States’ responses - Country Report Sweden. http://www.emnsweden.se/download/18.4cb46070161462db113232a/1524049376523/EMN-study_2017_The-changing-influx-of-asylum-seekers-in-2014-2016_SWEDEN_FINAL.pdf.

[3] Annamalai A. (2014). *Refugee health care: an essential medical guide*.

[4] Pooremamali P., Persson D., Eklund M. (2011). Occupational therapists' experience of working with immigrant clients in mental health care. *Scandinavian Journal of Occupational Therapy*.

[5] Taylor-Ritzler T., Balcazar F. E., Suarez-Balcazar Y., Kilbury R., Alvarado F., James M. (2010). Engaging ethnically diverse individuals with disabilities in the vocational rehabilitation system: themes of empowerment and oppression. *Journal of Vocational Rehabilitation*.

[6] Taylor-Ritzler T., Balcazar F., Dimpfl S., Suarez-Balcazar Y., Willis C., Schiff R. (2008). Cultural competence training with organizations serving people with disabilities from diverse cultural backgrounds. *Journal of Vocational Rehabilitation*.

[7] Balcazar F. E., Suarez-Balcazar Y., Taylor-Ritzler T. (2009). Cultural competence: development of a conceptual framework. *Disability and Rehabilitation*.

[8] Suarez-Balcazar Y., Rodawoski J., Balcazar F. (2009). Perceived levels of cultural competence among occupational therapists. *American Journal of Occupational Therapy*.

[9] Calzada E., Suarez-Balcazar Y. (2014). Enhancing cultural competence in social service agencies: a promising approach to serving diverse children and families. OPRE report. https://www.acf.hhs.gov/opre/resource/enhanching/cultural-competence-in-social-service-agencies-a-promising-approach-to-serving-diverse-children-and-families.

[10] Holstein J., Liedberg G. M., Öhman A., Kjellberg A. (2019). Validity and utility of the Swedish version of the cultural competence assessment instrument. *British Journal of Occupational Therapy*.

[11] Suarez-Balcazar Y., Baicazar F., Taylor-Ritzier T. (2011). Development and validation of the cultural competence assessment instrument: a factorial analysis. *Journal of Rehabilitation*.

[12] SFS 2003:460.Swedish Code of Statutes. The Act concerning the Ethical Review of Research Involving Humans. https://www.riksdagen.se/sv/dokument-lagar/dokument/svensk-forfattningssamling/lag-2003460-om-etikprovning-av-forskning-som_sfs-2003-460.

[13] World Medical Association Declaration of Helsinki (2013). Ethical principles for medical research involving human subjects. *JAMA*.

[14] Polit D. F., Beck C. T. (2004). *Nursing Research: Principles and Methods*.

[15] Polit D. F., Beck C. T. (2008). *Nursing research: generating and assessing evidence for nursing practice*.

[16] Field A. P. (2018). *Discovering statistics using IBM SPSS statistics*.

[17] Mundfrom D. J., Shaw D. G., Ke T. L. (2005). Minimum sample size recommendations for conducting factor analyses. *International Journal of Testing*.

[18] Bollen K. A. (1989). *Structural equations with latent variables*.

[19] Dragioti E., Wiklund T., Alföldi P., Gerdle B. (2015). The Swedish version of the insomnia severity index: factor structure analysis and psychometric properties in chronic pain patients. *Scandinavian Journal of Pain*.

[20] Goodwin L. (1999). The role of factor analysis in the estimation of construct validity. *Measurement in Physical Education and Exercise Science*.

[21] Polit D. F., Beck C. T. (2016). *Nursing research: generating and assessing evidence for nursing practice*.

[22] Brown T. A. (2015). *Confirmatory factor analysis for applied research*.

[23] Ferrans C. E., Powers M. J. (1992). Psychometric assessment of the quality of life index. *Research in Nursing & Health*.

[24] Darawsheh W., Chard G., Eklund M. (2015). The challenge of cultural competency in the multicultural 21st century: a conceptual model to guide occupational therapy practice. *The Open Journal of Occupational Therapy*.

[25] Beagan B. L. (2015). Approaches to culture and diversity: a critical synthesis of occupational therapy literature. *Canadian Journal of Occupational Therapy*.

[26] Taff S. D., Blash D. (2016). Diversity and inclusion in occupational therapy: where we are, where we must go. *Occupational Therapy in Health Care*.

[27] Kjellberg A., Kåhlin I., Haglund L., Taylor R. R. (2011). The myth of participation in occupational therapy – reconceptualizing a client-centred approach. *Scandinavian Journal of Occupational Therapy*.

[28] Taylor R. R. (2008). *The intentional relationship model: occupational therapy and use of self*.

[29] Bonder B. R., Martin L., Miracle A. W. (2004). Culture emergent in occupation. *American Journal of Occupational Therapy*.

[30] Taylor R. R. (2017). *Kielhofner's model of human occupation: theory and application*.

[31] Townsend E. A., Polatajko H. J. (2013). *Enabling occupation II: advancing an occupational therapy vision for health, well-being & justice through occupation: 9^th^ Canadian Occupational Therapy Guidelines*.

[32] Hultsjö S., Hjelm K. (2005). Immigrants in emergency care: Swedish health care staff’s experiences. *International Nursing Review*.

[33] Grandpierre V., Milloy V., Sikora L., Fitzpatrick E., Thomas R., Potter B. (2018). Barriers and facilitators to cultural competence in rehabilitation services: a scoping review. *BMC Health Services Research*.

[34] Hadziabdic E., Safipour J., Bachrach-Lindström M., Hultsjö S. (2016). Swedish version of measuring cultural awareness in nursing students: validity and reliability test. *BMC Nursing*.

[35] Holstein J. (2019). *Cultural competence for health professionals instrument development*.

